# Dr. Jekyll and Mr. Hyde: The Two Faces of the FUS/EWS/TAF15 Protein Family

**DOI:** 10.1155/2011/837474

**Published:** 2010-12-09

**Authors:** Heinrich Kovar

**Affiliations:** Children's Cancer Research Institute, St. Anna Kinderkrebsforschung, 1090 Vienna, Austria

## Abstract

FUS, EWS, and TAF15 form the FET family of RNA-binding proteins whose genes are found rearranged with various transcription factor genes predominantly in sarcomas and in rare hematopoietic and epithelial cancers. The resulting fusion gene products have attracted considerable interest as diagnostic and promising therapeutic targets. So far, oncogenic FET fusion proteins have been regarded as strong transcription factors that aberrantly activate or repress target genes of their DNA-binding fusion partners. However, the role of the transactivating domain in the context of the normal FET proteins is poorly defined, and, therefore, our knowledge on how FET aberrations impact on tumor biology is incomplete. Since we believe that a full understanding of aberrant FET protein function can only arise from looking at both sides of the coin, the good and the evil, this paper summarizes evidence for the central function of FET proteins in bridging RNA transcription, processing, transport, and DNA repair.

## 1. Introduction

The* Strange Case of Dr. Jekyll and Mr. Hyde, *a novel by the Scottish poet Robert Luis Stevenson (1850–1894), screened multiple times worldwide, describes the struggle between the good and evil sides of one individual [1]. At daylight, Dr. Jekyll is an honorable member of the society, but when the light fades he turns into an evil beast. The coexistence of two faces of one individual has inspired more than poetry and psychology. The question of which circumstances favor the surfacing of one or the other and how it may be influenced is relevant to all areas of life, including economy, technology and medicine. Cancer unravels the “Hyde” side of genes and their biology, but we can learn about how to tame fierce Mr. Hyde by understanding the Dr. Jekyll behind, the normal function of cancer genes.

FET (FUS, EWS, TAF15) proteins are a ubiquitously expressed family of similarly structured proteins predominantly localizing to the nuclear [2]. *FET* genes have attracted broad attention since all known members are found involved in deleterious genomic rearrangements with transcription factor genes in a variety of human sarcomas and acute leukemias. Chimeric FET proteins are considered and mostly studied as aberrant transcription factors. This paper aims at summarizing the good sides of FET proteins and looking at the characteristics of aberrant FET proteins as Dr. Jekyll's second face which surfaces only upon gene rearrangement or mutation.

## 2. Dr. Jekyll

### 2.1. The FET Family of Proteins

**Figure 1:**
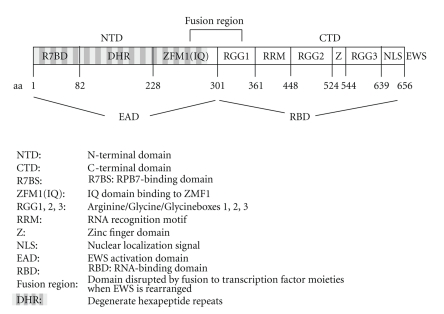
Structure of the prototype FET protein EWS.

The prototype FET protein EWS was identified in 1992 as the gene product encoded by the Ewing's sarcoma breakpoint region 1 (*EWSR1*) on chromosome 22q12 constituting the first identified member of a family of putative RNA-binding proteins [3], including also FUS/TLS/Pigpen/hnRNP P2 [4–7], TAF15/hTAF_II_68/TAF2N/RPB56 [8, 9], and Drosophila Cabeza/SARFH [10, 11] that share distinct structural characteristics (Figure 1). This protein family is frequently referred to as the FET (previously TET) (FUS/TLS, EWS, TAF15) family of proteins. Our restricted knowledge about the molecular functions of FET proteins derives mainly from protein interaction studies which identified more than 30 associated proteins mostly as part of protein/RNA complexes [12] (Table 1). Of note, pull-down experiments using EWS as bait revealed that all three FET proteins interact with each other and are therefore likely to be part of the very same protein complexes. As demonstrated for EWS, the association with most interacting proteins depends on the presence of RNA and is destroyed upon RNaseA treatment (Table 1). The functional roles of interacting proteins suggest a general bridging role for FET proteins coupling RNA transcription, processing, transport, and DNA repair.

### 2.2. RNA Binding of FET Proteins

Several functional FET domains were defined (see Figure 1): the N-terminal domain is largely composed of a highly repetitive primary sequence containing multiple copies of a degenerate hexapeptide repeat motif similar to the C-terminal domain of RNA polymerase II. The C-terminal domain (CTD) contains a conserved nuclear import and retention signal (C-NLS) [13], a putative zinc-finger domain, and a conserved RNA recognition motif (RRM) flanked by 3 arginine-glycine-glycine (RGG) boxes [14] compatible with RNA binding of FET proteins. FUS has been demonstrated to bind preferentially to GGUG-containing RNAs [15]. EWS might have similar sequence specificity since it was demonstrated to bind strongly to both poly G and poly U, but not to poly A and poly C RNA, homopolymers [16]. Although it is only the zinc finger domain of FUS that makes physical contact with the GGUG motif, all three RGG boxes together with the RRM contribute to this activity [15]. Intriguingly, a recent study identified strong binding of FUS to human telomeric RNA [17] and to small low-copy-number RNAs tethered to the promoter of cyclin D1 [18]. Nothing is known about the RNA binding specificity TAF15.

### 2.3. A Role for FET Proteins in RNA Transcription

The FET N-terminal domain (NTD) resembles the activation domain of certain transcription factors such as SP-1 rich in glutamine and proline residues. When fused to a DNA-binding domain (DBD), as is the case in oncogenic FET derivatives, the NTD strongly activates reporter gene activity in a DNA-binding-dependent way [19–23]. The critical determinants for this transactivation activity are dispersed throughout the NTD [24], which is intrinsically disordered [25]. It is comprised of a variable number of a degenerate hexapeptide repeat motif (DHR) with the consensus SYGQQS, with homologies to the C-terminus of RNA polymerase II [3]. Mutation analysis of the EWS NTD revealed a critical dependence of the transactivation activity on the aromatic side chain of the conserved tyrosine residue present in the DHR [21].

The function of the NTD in the context of germline FET proteins remains largely unexplored. When included into artificial FET-DBD fusion proteins, the CTD inhibited transcriptional activation by the NTD [26]. More recent data demonstrated that the RGG motifs of the FET-CTD repress a range of transcriptional activation domains [27]. The context-dependent difference in the transactivation potential of the NTD might be explained by different structures and accessibility of the NTD for protein interactions in the presence and absence of the CTD [28]. Protein interaction between the very N-terminus of EWS and the RNA PolII holoenzyme component hsRPB7 was only observed for EWS-FLI1 and C-terminal truncated EWS, while interaction with hsRPB5 and hsRPB3 was restricted to germline EWS [29, 30]. EWS has been reported to support CREB-binding-protein-(CBP/p300-) dependent activation by the transcription factors HNF-4 and OCT-4 [31, 32] which is inhibited by the EWS-interacting protein STRAP (serine-threonine kinase receptor-associated protein) [33]. Similarly, FUS acts as a positive cofactor for NFkappaB-mediated transcription [34]. In contrast, EWS repressed BRN3A-dependent transcription [35].

All three FET proteins were found to associate with RNA polymerase II and subpopulations of the TF_II_D complex, respectively[8, 29, 36]. Consistent with an evolutionary conserved role of FET proteins in RNA transcription, SARFH was found to be associated with transcribed chromatin in Drosophila [10]. Interactions of the NTD with various transcription factors were described (FUS with steroid, thyroid hormone, and retinoid receptors [37], EWS with Brn3A and via CBP/p300 with HNF4 and OCT4 [31, 32, 35, 38]). Interestingly, EWS and FUS were found to bind directly to the proximal elements of the macrophage-specific promoter of the CSF-1 receptor (*CSF1R*) gene and also to high-affinity sites recognized by myeloid zinc finger protein 1 (Mzf1) suggesting a role in transcriptional start site selection of TATA-less promoters [39].

Besides their role in RNA-polymerase-II-mediated transcription, the recent finding of FUS repressing RNA polymerase III-dependent transcription of small untranslated RNAs implies a more general role for FET proteins in the orchestration of the transcriptome [40].

### 2.4. A Role for FET Proteins in mRNA Maturation

The RNA-binding specificity of FUS for the GGUG motif found in 5′splice sites suggests a role in RNA processing. EWS and FUS were identified within the same RNA-splicing complex together with polypyrimidine-tract-binding-protein-associated factor (PSF) [41]. In addition, EWS and FUS associate with a variety of splicing factors such as U1C, SR, SF1, and YB1 [15, 42–47]. Further, EWS NTD and FUS bind to novel RNA helicases [48, 49]. Moreover, interaction of the EWS NTD with BARD1, a protein playing an important role in the inhibition of RNA maturation at sites of stalled transcription upon DNA damage, was reported [31, 50–53]. Together, these results suggest that FET proteins couple RNA transcription to processing. The mechanism and specificity of this activity remain largely unknown.

### 2.5. A Role for FET Proteins in the Processing of Small Noncoding RNAs

EWS was recently identified in a protein complex with the nuclear RNase III DROSHA [54]. While DROSHA is known to be central to the cleavage of the pre-micro-RNA (miRNA) precursor from the primary miRNA transcript thereby initiating miRNA processing and transport to the cytoplasm, evidence for a functional role of the EWS containing DROSHA complex is missing. Therefore, a general role for EWS in the metabolism of noncoding RNAs remains to be demonstrated. Since about a quarter of miRNA genes are encoded in the introns of protein-coding genes [55], it is intriguing to speculate that EWS links not only transcription to RNA splicing but also to the generation of miRNAs from gene introns. This, so far hypothetical, activity may gain importance in the light of frequent negative posttranscriptional regulation of miRNA processing at the DROSHA level in cancer [56].

### 2.6. A Role for FET Proteins in RNA Transport

Consistent with their proposed function in gene regulation, FET proteins are mostly nuclear, localizing to inclusions such as the coiled body and the nucleolus (demonstrated for EWS, FUS, and pigpen in [9, 57, 58]). There is also evidence that FET proteins shuttle between the nucleus and the cytoplasm raising the possibility that they play a role in RNA transport [59, 60]. In mouse hippocampal neurons, FUS is localized to neuronal dendrites and, upon activation, translocate to the spines, where local translation takes place, carrying along specific mRNA transcripts [61, 62]. This finding implicates FET proteins in localizing cytoplasmic determinants for the local control of protein synthesis and secretion, at least in neurons.

For EWS, RNA binding, subcellular localization, and consequently transcriptional activity have been found to be regulated by extensive asymmetric dimethylation of the RGG motifs, mediated by protein arginine methyltransferases 1 and 8 (PRMT1, PRMT8) [67–70], which likely impacts on self-association of intact EWS required for nuclear localization [71, 72]. Extensively methylated EWS has even been identified on the cell surface [73]. So far, the functional relevance of these findings has yet to be determined.

### 2.7. A Role for FET Proteins in Genome Surveillance and DNA Repair

FUS deficiency in mice resulted in defective B-lymphocyte development and activation, high levels of chromosomal instability, and perinatal death [74]. EWS knock-out mice also displayed disrupted B-cell development and were extremely sensitive to ionizing radiation. Together with a defect in homologous recombination impairing meiosis and the observation of premature senescence of embryonic fibroblasts, these results suggest a role for EWS in recombination repair [75]. In the zebrafish, silencing of *EWS* genes during embryogenesis led to mitotic defects followed by p53-dependent apoptosis [76].

Consistent with the phenotype of FET deficiency in genetically modified mice, the interaction of EWS (and EWS-FLI1) with the BRCA1-associated ring finger domain protein BARD1 may point to a role of FET proteins in DNA double-strand break repair [53]. This hypothesis is strengthened by high genomic instability in FUS knock-out mice [74] and radiation sensitivity and impaired homologous recombination in EWS knockouts [75]. The recently discovered homologous DNA-strand-pairing activity of all four FET proteins may functionally contribute to this role [77].

Intriguingly, the RNA binding activity of FUS was reported to act as a sensor for DNA damage and to elicit transcriptional repression; as exemplified for cyclin D (*CCND1*) promoter regulation, DNA damage was demonstrated to induce the expression of single-stranded, low-copy-number ncRNA transcripts tethered to the 5′ regulatory regions of *CCND1* which recruit FUS and allosterically modify it to bind to and repress CREB-binding protein (CBP) and p300 histone acetyltransferase activities [18].

Activation of gene transcription by many, if not all, sequence-specific transcription factors requires DNA-topoisomerase-II-beta-dependent, transient, site-specific dsDNA break formation [78]. One may speculate that the proposed role of FET proteins in recombination repair is linked to their association with transcription initiation complexes at promoter regions.

## 3. Mr. Hyde

### 3.1. The Role of FUS in Neurodegenerative Disease

Point mutations of FUS have recently been found in a subset of patients with familial amyotrophic lateral sclerosis (ALS), a neurodegenerative disorder destroying motoneurons [79, 80]. Previously, this disease has been associated with mutations in either superoxide dismutase 1 (SOD1) or TDP43 (43 kDa TAR DNA-binding domain protein). TDP43 is an essential nuclear RNA-binding protein that participates in transcriptional repression, exon splicing inhibition, and mRNA stabilization. The convergent phenotypes associated with FUS and TDP43 mutations suggest that they are part of the same machinery. In fact, TDP-43 and FUS were demonstrated to function in a biochemical complex to modulate expression of HDAC6, a recently identified mRNA substrate of TDP-43 [81]. 

### 3.2. The Oncogenic Function of FET Fusion Protein

The predominant type of *FET* gene aberrations is that of fusions to various transcription factor genes by which the FET RNA-binding domain is replaced by the DNA-binding domain of the transcription factor (Table 2). FET fusion proteins are capable of transforming cells in culture dependent on the cellular context. EWS-ETS fusions, for example, transform NIH3T3 and bone-marrow-derived mesenchymal progenitor cells, but not human or rat primary fibroblasts, mouse embryonic stem cells, or embryonic fibroblasts [102, 103]. The phenotype of tumors obtained in immunodeficient mice after transplantation of EWS-ETS-transformed NIH3T3 cells clearly differs from that obtained after transformation with other EWS-transcription factor fusions and resembles that of Ewing's sarcoma [104, 105]. In the xenograft model, the amino terminal portion of EWS, as well as FUS (and presumably also TAF15), is functionally interchangeable in the fusion protein, while the transcription factor moiety determines the tumor phenotype [7]. Functional interchangeability of the FET-NTD is also reflected in human sarcomas: both *EWS-CHOP* and *FUS-CHOP* characterize myxoid liposarcoma [5, 94], and *EWS-NR4A3* and *TAF15-NR4A3* are found in extraskeletal myxoid chondrosarcoma [106]. It was therefore hypothesized that FET fusion proteins affect differentiation programs by aberrant regulation of genes specifically recognized by the transcription factor DNA-binding moiety.

The best studied example in this respect is EWS-FLI1 in Ewing's sarcoma family tumors (ESFT). Using experimental knockdown of EWS-FLI1 in ESFT cell lines and comparison to primary tumours and normal tissues, signatures of the chimeric transcription factor on the ESFT transcriptome were defined [125, 130, 131]. An almost equal number of genes were found activated and repressed by EWS-FLI1. Of the approximately 600 to 800 significantly dysregulated genes, only a fraction is directly bound by EWS-FLI1 and many EWS-FLI1 bound genes do not show aberrant regulation (our unpublished observations). Over the years a number of directly EWS-FLI1-regulated genes have been characterized in ESFT (Table 3). It is interesting to note that almost all attempts to experimentally restore the presumed “normal” expression pattern of these targets in ESFT cell lines (by ectopic reexpression of EWS-FLI1 repressed genes and knockdown of EWS-FLI1-activated genes) resulted in reduced tumor cell growth in vitro and/or reduced tumorigenicity in vivo and in several cases enhanced chemosensitivity (Table 3). These results suggest that directly EWS-FLI1-regulated genes play essential roles in the establishment and/or the maintenance of the malignant phenotype of ESFT.

Functional annotation of EWS-FLI1-regulated genes revealed that activated genes primarily annotate to proliferation-associated functions, while genes involved in developmental and differentiation processes are predominantly repressed [131], suggesting that EWS-FLI1 suppresses differentiation of the enigmatic ESFT precursor cell. In fact, sustained silencing of EWS-FLI1 restores the potential of ESFT cells to differentiate along adipogenic, neuronal, and osteogenic lineages [132], a feature shared with mesenchymal stem cells (MSC). Conversely, ectopic EWS-FLI1 expression blocks the differentiation potential of MSC and imposes an ESFT-like phenotype on them [103, 133, 134]. Consistent with the role of EWS-FLI1 in the disruption of developmental differentiation processes is the finding of skeletal malformations in mice expressing transgenic EWS-FLI1 in the mesenchymal lineage [135]. Similarly, the FUS-ERG fusion found in human myeloid leukemia with the t(16;21) translocation was demonstrated to block terminal differentiation of and confer a growth advantage to human myeloid progenitor cells [136].

Consistent with early in vitro data [19–21], activated genes showed an enrichment of ETS-binding motifs in their promoters while this motif was underrepresented in repressed genes [131]. This result suggests that gene repression regulating differentiation genes might be mediated by indirect mechanisms. One such mechanism involved in blocking osteogenic differentiation is interaction and interference of EWS-FLI1 with the master regulator of bone and cartilage development, RUNX2 [137]. RUNX2 was demonstrated to bind also to intact EWS and FUS [138]. A number of different transcription factor binding motifs overrepresented in the promoters of EWS-FLI1-repressed genes may be indicative of other protein interactions that remain to be defined. Additional mechanisms of gene repression downstream of EWS-FLI1 involve the activity of transcriptional repressors whose expression is upregulated by EWS-FLI1 such as NKX2.2 [139] or the epigenetic modifier EZH2 [120] and the regulation of microRNAs [140]. An alternative intriguing mechanism may involve the binding of EWS-FLI1 to microsatellites outside of promoter regions even at distances of several megabases from the transcriptional start sites [141–143]. While these elements can activate transcription when juxtaposed to a promoter, their activity and mechanism of action from distant sites remains elusive.

Interestingly, there is evidence that EWS and EWS-FLI1 form a fatal liaison in that genes targeted by the FLI1 DNA-binding domain encode for proteins that interact with the EWS N-terminal domain in both the intact EWS protein and the chimeric protein. This is the case for NR0B1, a protein known to form large complexes with the stem cell factors OCT3 and OCT4, as well as EWS [32, 144, 145]. Intriguingly, the translocation t(6;22)(p21;q12) found in some undifferentiated sarcomas and neoplasms of skin and salivary glands directly fuses *OCT4* to *EWSR1*. Among EWS-FLI1-repressed genes is also hsa-mir-145, a microRNA targeting OCT4 and other stem cell factors and feeding back on EWS-FLI1 expression [133]. These findings provide evidence that EWS and EWS-FLI1 form a functional network in the regulation of tumor cell stemness.

### 3.3. A Transcription-Independent Role for the EWS-FLI1 Fusion Protein

The first indication that malignant transformation by FET fusion proteins may involve functions other than direct transcriptional activation of target genes recognized by the DBD came from functional dissection of the EWS-FLI1 fusion protein in NIH3T3 transformation assays. These studies suggested that the minimal transforming and the minimal transcriptional activation domains can be separated from each other [146]. Specifically, the 83 N-terminal amino acids were sufficient to transform NIH3T3 cells when fused to the FLI1 DBD. Protein interactions with this domain were found to be context dependent [28–30]. In addition, residual transforming activity of EWS-FLI1 was retained even when the FLI1-DBD was destroyed, suggesting a DNA-binding-independent function for the oncogenic fusion protein [147, 148]. Also, EWS-FLI1 was shown to inhibit the CBP-dependent transcriptional activity of the retinoid acid (RA) receptor RXR desensitizing cells to the differentiation and apoptosis inducing activity of RA by a mechanism unrelated to DNA binding [38].

Protein interaction studies revealed that the EWS-NTD and the FUS-NTD in the context of their oncogenic fusion proteins communicate with the same RNA processing factors as in germline EWS [42–44, 46, 47, 53] but interfere with serine arginine protein (SR) and YB1-mediated splicing [42, 44, 45]. In addition, it was demonstrated that EWS-FLI1, but not EWS, interfered with heterogeneous nuclear ribonucleoprotein A1-dependent 5′ splice site selection in an in vivo E1A splicing assay [149]. This result might possibly be explained by a dominant negative effect of EWS-FLI1 on the RNA processing function of EWS that remains to be investigated. In fact, we have previously demonstrated that EWS-FLI1 can interact with its germline counterpart [64]. Importantly, mutational analysis of EWS-FLI1 revealed that the ability to affect pre-mRNA splicing coincided with transforming activity [149]. These results suggest a role for EWS-FLI1 in RNA processing. However, this role may not be regarded as transcription independent. A recent study of transcriptional elongation of the direct EWS-FLI1 target gene cyclin D1 (*CCND1*) revealed that both EWS and EWS-FLI1 stimulate transcription of the gene, but elongation by EWS-FLI1 is significantly slowed down in comparison to EWS. As a result, expression of the oncogenic splice isoform D1b is favoured over the splice isoform D1a [150]. So far it remains unknown how many genes may be affected by this or a similar phenomenon.

### 3.4. EWS-FLI1 and Disrupted Tumor Suppression

FET fusion proteins are aberrantly expressed transcription factors driving cell proliferation. As such they impose oncogenic stress on the cell triggering the p53 checkpoint [151]. ESFT escape the oncogenic stress imposed by EWS-FLI1 by modulating p53 activity. Two mechanisms for this oncogenic property of EWS-FLI1 have recently been described: interference with tumor suppressive NOTCH signalling pathway activity through transcriptional regulation of autocrine NOTCH ligand expression [152] and direct interaction with p53 [153]. It should be noted, however, that the ability of EWS-FLI1 to modulate p53 activity is tissue dependent. In fibroblasts, EWS-FLI1 was demonstrated to elicit a p53-mediated cell-cycle arrest [151]. Most other cell types do not tolerate EWS-FLI1 expression at all and die in response to ectopic expression of the chimeric oncogene (for review [102]). The only tissue permissive to the oncogenic properties of EWS-FLI1 identified so far is mesenchymal stem cells [103]. The tissue-specific factors that steer the p53 response into the one (growth arrest/apoptosis) or the other (escape from oncogenic stress) direction remain to be elucidated.

There is also evidence for EWS-FLI1 interfering with the other central tumor suppressor pathway in oncogenesis: although the mechanism still remains to be defined, knockdown of EWS-FLI1 in ESFT cells leads to pRB-1 hypophosphorylation [154].

## 4. Getting Hold of Mr. Hyde

The development of small molecule inhibitors of biological macromolecules, originally in the context of chromosome translocations, has been pioneered by research on receptor tyrosine kinases. Here, the design of smart molecules is guided primarily by crystallography and structure/function analyses of the target proteins. For FET fusion proteins, this approach is not feasible because of the intrinsic disorder of their structure. However, recent landmark studies provided proof of principle for successful interference with protein interactions of intrinsically disordered proteins [155]. Guided by a peptide aptamer screen, a small molecule mimetic was described that competes with RNA helicase A for interaction with the EWS N-terminus in the context of the EWS-FLI1 fusion protein and slowed tumor formation in mice [156]. There is evidence from protein interaction studies that the faces of the EWS N-terminus look different in the context of the wildtype protein (Dr. Jekyll) and the transcription factor fusion protein (Mr. Hyde) [28–30]. Thus, there is hope that the evil culprit for the development and progression of several sarcomas and leukemias that is still hiding in the dark can be successfully targeted in the near future.

## Figures and Tables

**Table 1:** EWS interacting proteins: *not bound by methylated EWS; **not bound by methylated EWS upon RNaseA treatment.

RNase A sensitive	
hnRNP A0 [12]	Pre-mRNA processing, RNA metabolism, RNA transport
hnRNP A1 [12]	Pre-mRNA processing, RNA metabolism, and RNA transport may modulate splice site selection
hnRNP A2B1 [12]	Pre-mRNA processing, RNA metabolism, RNA transport
hnRNP A3 [12]	Regulation of age-related gene expression, binds to telomeric RNA
hnRNP A/B [12]	Binds to multiprotein editosome complex
hnRNP A18* [12]	Stabilization of transcripts, genotoxic stress response, translational activator, binds to 3′UTR
hnRNP D0 [12]	Regulation of mRNA stability
hnRNP F [12]	Binds G-rich sequences
hnRNP G [12]	Regulation of splice site selection, DNA double-strand break repair
hnRNP H [12]	Pre-mRNA alternative splicing regulation
hnRNP H2 [12]	Involved in Fabray disease and X-linked agammaglobulinemia
hnRNP H3 [12]	Early heat shock-induced splicing arrest
hnRNP Q [12]	RNA stability, translationally coupled mRNA turnover
Small nuclear ribonucleoprotein Sm D3 [12]	Pre-mRNA splicing and small nuclear ribonucleoprotein biogenesis, histone 3′-end processing
U1 small nuclear ribonucleoprotein A* [12]	First snRNP to interact with pre-mRNA for the subsequent binding of U2 snRNP and the U4/U6/U5 tri-snRNP
Splicing factor, arginine/serine-rich 1* [12, 44]	Accuracy of splicing and regulation of alternative splicing
Splicing factor, arginine/serine-rich 3* [12]	Putative proliferation-/maturation-associated RNA processing
Splicing factor, arginine/serine-rich 9* [12]	Constitutive splicing
RRM containing coactivator activator [12]	Activation/modulation of nuclear receptors
Tubulin alpha ubiquitous chain [12]	Scaffold for cell shape and organelle movement
Vimentin^1^	Organizer of a number of critical proteins involved in attachment, migration, and cell signaling

RNase insensitive	

Protein arginine N methyltransferase 1 [12]	Epigenetic regulation, signal transduction, DNA repair
Protein arginine N-methyltransferase 8 [63]	Localized at cell membrane
hnRNP M [12]	Splicing, selective recycling of immature GlcNAc-bearing, thyroglobulin molecules, potentially involved in signalling
hnRNP U [12, 43]	Binds double- and single-stranded RNA and DNA, binds pre-mRNA
FUS** [12]	This review
TAF15* [12]	This review
EWS* [64]	This review
RNA-dependent helicase p68 (DDX5) [12]	RNA-dependent ATPase, alteration of RNA secondary structure in splicing and translation initiation
RNA-dependent helicase p72 (DDX17) [12]	RNA-dependent ATPase, alteration of RNA secondary structure in splicing, and translation initiation
ATP-dependent RNA helicase A [12]	ATP-dependent unwinding of double-stranded RNA and DNA-RNA complexes, transcriptional regulation
ATP-dependent RNA helicase DHX36* [12]	Deadenylation and decay of mRNAs with 3′-UTR AU-rich elements
Elongation factor EF1 gamma [12]	Translation elongation, role in anchoring the translational complex to other cellular components
Elongation factor EF1 alpha [12]	Translation elongation, promotes aminoacyl-tRNA binding to ribosome
Dead box protein 3 X (DDX3X)* [12]	ATP-dependent RNA helicase
Tubuline beta-2 chain [12]	Scaffold for cell shape and organelle movement

RNA dependence unknown:	

RBP3 [29]	RNA Polymerase II component
TAF5 [29]	General transcription factor TF_II_D component
TAF7 [29]	General transcription factor TF_II_D component
TAF11 [29]	General transcription factor TF_II_D component
TAF13 [29]	General transcription factor TF_II_D component
Brn-3a [35]	Transcription factor
SF1 [47]	Splicing factor
YB1 [42]	Splicing factor
Survival motor neuron protein* [65]	Essential role in spliceosomal snRNP assembly in the cytoplasm and is required for pre-mRNA splicing in the nucleus
Serine threonine kinase receptor (STRAP) [33]	Inhibits transforming growth factor beta (TGF-beta) signaling
BARD1 [53]	DNA repair, mRNA maturation
Pyk2 [66]	Tyrosine kinase, signal transduction

**Table 2:** FET gene fusions in cancer. TF: transcription factor.

Phenotype	FET partner	TF partner	TF type	Ref.
ESFT				
(85%)	EWS	FLI1	ETS	[3]
(10%)	EWS	ERG	ETS	[82, 83]
(1%)	EWS	ETV1	ETS	[84]
(1%)	EWS	ETV4	ETS	[85, 86]
(1%)	EWS	FEV	ETS	[87]
(1%)	FUS	FEV	ETS	[25]
(1%)	FUS	ERG	ETS	[88]
ESFT-like	EWS	NFATC2	rel related	[89]
Askin-like, CD99 neg.	EWS	ZNF278	zinc finger	[90]
Bone sarcoma	EWS	POU5F1	pou	[91]
Mucoepidermoid carcinaoma	EWS	POU5F1	pou	[92]
Hidradenoma	EWS	POU5F1	pou	[92]
	EWS	PBX1	homeobox	[92]
Low-grade fibromyxoid sarcoma	FUS	CREB3L1	Leucine zipper	[93]
Myxoid liposarcoma	EWS	DDIT3	bZIP	[94]
	FUS	DDIT3	bZIP	[5]
Clear cell sarcoma	EWS	ATF1	bZIP	[95]
	EWS	CREB1	bZIP	[96]
Desmoplastic SRCT	EWS	WT1	zinc finger	[97]
Extraskeletal myxoid chondrosarcoma	EWS	NR4A3	nuclear receptor	[98]
	TAF15	NR4A3	nuclear receptor	[99]
AML	FUS	ERG	ETS	[100]
cALL, AUL	EWS	ZNF384	zinc finger	[101]
AML, ALL	TAF15	TAF15	zinc finger	[101]

**Table 3:** Validated direct EWS-FLI1 target genes.

EWS-FLI1 activated genes	Consequences of target suppression
*Id2* [107]	*Not known*
*GLI1* [108]	Reduced anchorage independent growth [109]
VEGF [110]	Decreased osteolysis [111]
*STYXL1 *[112]	Not known
*PLD2* [113]	Inhibition of PDGF BB signalling
*PTPL1 *[114]	Reduced growth and increased chemosensitivity [114]
*CAV1* [115]	Reduced anchorage independent growth, reduced tumorigenicity
*GSTM4* [116]	Abrogation of oncogenic transformation, increased chemosensitivity [116]
*NR0B1* [117, 118]	Abrogation of oncogenic transformation [119]
*EZH2 *[120]	Reduced anchorage independent growth, reduced tumorigenicity [120]
*AURKA, AURKB *[121]	Not known
*Tenascin C *[122]	Not known

EWS-FLI1 repressed genes	Consequences of target restoration

*TGFBR2* [123]	Loss of tumorigenicity [123]
*CDKN1A* [124]	Inhibition of cell growth [124]
*IGFBP3* [125]	Inhibition of cell growth and motility [126]
*FOXO1 *[127]	Not known
*DKK1 *[128, 129]	Decreased tumorigenicity [128]
